# Non‐invasive urine markers for the differentiation between RCCs and oncocytoma

**DOI:** 10.1002/jcla.23762

**Published:** 2021-05-07

**Authors:** Melanie von Brandenstein, Jan Herden, Barbara Köditz, Manuel Huerta, Tim Nestler, Axel Heidenreich, Jochen W.U. Fries

**Affiliations:** ^1^ Department of Urology Faculty of Medicine and University Hospital Cologne University of Cologne Cologne Germany

**Keywords:** ELISA, kidney cancer, Mxi‐2, small renal cancer, urine biomarker, Vim3

## Abstract

**Background:**

Recently, our group showed that Vim3 is overexpressed in tissue samples of renal oncocytomas and Mxi‐2 in clear cell renal carcinoma (ccRCC). The mechanism leading to the truncation of both proteins is known and involves with two miRs, both detectable in urine. Since the analysis of miRs is time‐consuming, our aim was to identify the truncated proteins in urine instead. Furthermore, urine samples from small renal masses (SRMs) (n = 45, <4 cm) were analyzed to get a pre‐surgical differentiation of the cancer subtypes.

**Methods:**

Urines were accessed from the urological biobank (n = 350). Proteins were isolated from urine samples, and Western blots were performed. Each sample was analyzed with ELISA for the expression of Vim3 and Mxi‐2. A lateral flow assay was established. For the detection of SRMs, the miRs were isolated and qRT‐PCR was performed.

**Results:**

A significant increase of Vim3 in urines from patients with oncocytoma (n = 20) was detectable with ELISA compared to all other subtypes of RCCs (chromophobe (n = 50), papillary (n = 40), ccRCC (n = 200), and controls (n = 40) (***p < 0.0001)). Mxi‐2 was predominantly overexpressed in ccRCCs (***p < 0.0001). Lateral flow assay of Vim3 and Mxi‐2 shows two bands in the case of oncocytoma and ccRCC indicating the specificity of this test.

For SRMs, an overexpression of miR‐15a/Mxi2 was detectable in urine samples from ccRCC and chromoRCC patients. In contrast to that, miR‐498/Vim3 were predominantly overexpressed in oncocytoma patients.

**Conclusion:**

Both proteins (Vim3 and Mxi‐2) were detectable in patients’ urines and can be used for the non‐invasive differentiation of kidney cancers.

## Graphical abstract

In urine sample from clear cell renal carcinoma (RCC) and oncocytoma patients, two miRs (miR‐15a and miR‐498) were significantly overexpressed. This overexpression of the miRs leads to the production of two truncated proteins (Mxi‐2 and Vim3), both detectable in urine samples. We analyzed n = 350 urine samples from healthy patients and those with different urological tumors. The noticed overexpression of Mxi‐2 and Vim3 in urine samples significantly correlates with the tumor entity. Overexpression of Mxi‐2 in RCC samples and Vim3 in oncocytomas, also in small renal cancers (<4 cm), is possible even before surgery.

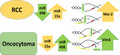

## 1 INTRODUCTION

Our group recently showed that due to miR (microRNA) binding on the DNA level, DNA transcription is stopped and results in C‐terminal truncated and intron sequence included proteins.^1^ Vimentin 3 (Vim3) is the truncated version of the full‐length vimentin and is produced by the binding of miR 498 to DNA. This in turn leads to a transcriptional stop and the production of Vim3, a protein with an unique C‐terminal ending.^1^ Under normal conditions, full‐length vimentin is responsible for and important in cell shape, structure, and anchorage.^2^ Since Vim3 misses the C‐terminal ending of the full‐length vimentin, a normal cell structure cannot be established, due to the missing tetramer formation of vimentin in these cells. As a result, more cell organelles are capable of an increased movement in the cytoplasm and a larger number of mitochondria can be present in the cell.^2^ An overexpression of mitochondria exists in benign renal oncocytoma; nevertheless, these mitochondria are without function. Increased Vim3 levels were detectable only in oncocytoma tissue samples compared to all other kidney tumor entities.^3^ However, due to this finding and the well‐known correlation between the miR‐498 and Vim3, our aim was to detect Vim3 in patients’ urine, since this is a non‐invasive method. Former studies could demonstrate that miR‐15a is overexpressed in urine samples from patients with RCC ^4^ and decreases to a normal miR‐15 level after tumor removal. miR‐15a is responsible for the truncation of MAPK p38α and also results in a C‐terminal truncated protein with a unique C‐terminal ending, called Mxi‐2.^1^ An increased expression of Mxi‐2 has already been shown to be detectable in urine samples in former studies. This led us to question whether a non‐invasive method to differentiate benign renal tumors and kidney cancer could be performed by analyzing patients’ urine for both miRs, namely miR‐498 and miR‐15a, in addition to the much cheaper and faster ELISA analysis of the target proteins (Vim3 and Mxi‐2). Furthermore, we collected urine samples from patients suffering from small kidney cancers (<4 cm) for pre‐surgical classification of the cancer.

## 2 MATERIALS AND METHODS

### 2.1 Patient collective

Between 2015 and 2018, urine samples (midstream urine) were collected (independent of the tumor size and location in the kidney) at the University Hospital of Cologne from each patient with a suspicion of kidney cancer. All samples were part of the urological biobank, and all patients signed the BioMASota formula permitting the use of their samples in research (as approved by the Ethics Committee of the Medical Faculty of the University of Cologne (file reference 12–163.

**TABLE 1:** Primer sequences

Gene	Sequence	Annealing temp.	No. of cycles
Mxi−2	Forw 5’‐GACTCAGATGCCGAAGAT−3’	50°C	40×
	Rev 5’‐TCAACTAATGGTACTTTATTTGG−3’		
Vim3	Forw. 5’‐GAGAACTTTGCCGTTGAAGC−3’	50°C	40×
	Rev. 5’‐GAAATAAAATGCTTACCCCTCAG−3’		
ß‐actin	Forw 5’‐TTGGCAATGAGCGGTTCCGCTG−3’	50°C	40×
	Rev 5’‐TACACGTGTTTGCGGATGTCCAC−3’		

The final cohort consisted of n = 350 patients with pathohistological proof of an oncocytoma (n = 20) or a RCC [chromophobe n = 50; papillary (n = 40); clear cell RCC (ccRCC) (n = 200)], as well as negative controls (n = 40). For detailed information of each group, Table 2.

**TABLE 2:** Patients collected for the different tumor groups showing the heterogeneity of the signal groups

Oncocytoma	Age (years)	Tumor size (cm)	Tumor grading
Female (n = 15)	61.5 ± 9.7	4.0 ± 1.7	
Male (n = 25)	63.1 ± 9.6	4.6 ± 2.7	
Chromophobe RCC			
Female (n = 24)	69.2 ± 5.1	4.3 ± 2.5	pT1a‐pT2b, pN0,M0, R0,V0,G2‐G3
Male (n = 26)	69.2 ± 9.1	4.57 ± 2.2	
Papillary RCC			
Female (n = 28)	70.33 ± 6.3	4.14 ± 1.78	pT1a‐pT2a, pN0,M0, R0,V0,G1‐G2
Male (n = 32)	66.7 ± 9.8	4.2 ± 2.2	
Clear cell RCC			
Female (n = 96)	64 ± 10.1	6.2 ± 3.6	pT1a−3a,pN0‐pN2, M0‐M1, R0, V0‐V1,G1‐G2
Male (n = 104)	67.71 ± 8.2	7.7 ± 3.0	

The group of small kidney cancers (n = 10) with a size of <4 cm and a regression rate <10% consists of n = 45 patients including controls (n = 20) from healthy donors. The regression rate <10% was of importance for over a regression about 10% PKCα increase again which decrease the expression of miR‐15a and therefore the Mxi‐2 expression as well.^4^

### 2.2 Western blot analysis

Western blot analysis was performed from 50 µl urine samples. Urine was centrifuged and washed twice with PBS, the sediment fragments were then incubated with ice‐cold RIPA buffer for 30 min on ice, and proteins were isolated. For the analysis of Mxi‐2, a commercially available antibody from nanoTools (clon 2F2) was used and tested for specificity with the provided positive control lysate (A431). For the Vim3 analysis, the already tested and published Vim3 antibody (Davids BioLab) was used.^3^ ß‐actin was applied according to the manufacturer's protocol and used for neutralization. All blots were done in triplicates and analyzed with INTAS Chemostar.

### 2.3 ELISA

ELISA plates were washed twice with 1xPBS and incubated with 3B4 Vimentin antibody (against the full‐length and truncated version) 1:500 for 1 h at room temperature. Afterward, wells were washed 2× in PBS and incubated with 50 µl patient urine at room temperature for 1 h. Wells were washed again with PBS 3× and incubated with Vim3 antibody overnight at 4°C. The ELISA plate was washed again with PBS and incubated with the mouse HRP‐labeled secondary antibody for 1 hour at room temperature. For Mxi‐2, ELISA plates were incubated with the non‐purified urine samples for 1 h at room temperature. Afterward the plates were washed 3× with PBS and incubated for 2 h at room temperature with the Mxi‐2 antibody. Finally, the plates were washed 3× with PBS. TMB was used according to the manufacturer's protocol, and all reactions were stopped after exactly 10 min with stopping solution. For ELISA analysis, the FLUOstar Omega reader was used.

### 2.4 Lateral flow assay

The lateral flow assay was performed as exemplified in Figure 4. HF 180 was used as analytic membrane. Antibodies were incubated overnight at room temperature and in the dark. As a conjugation pad, the GDFX membrane was used and incubated with the corresponding antibody for 3 h in a dry environment at 37°C. Finally, the lateral assay was set up and the urine sample (50 µl) was incubated for 10 min. For the analysis, fluorescent secondary antibody was used. All lateral flow assays were done in triplicates and analyzed with INTAS Chemostar.

### 2.5 Total RNA isolation from urine

For miR isolation from patients’ urine, 500 µl of urine was used and added to the QIAzol reagent, mixed, and further used according to the manufacturer's protocol (miRNeasy kit; Qiagen, Hilden, Germany). RNA quantification was accomplished using NanoDrop technology.^5^

### 2.6 cDNA synthesis

cDNA was obtained from 150 ng of RNA using random primers and SuperScript III reverse transcriptase, according to the manufacturer's protocol (Invitrogen, Darmstadt, Germany). The RT‐PCR was performed as previously described.^4^, ^5^

### 2.7 Quantitative real‐time PCR (qRT‐PCR)

1 µl of the cDNA (transcribed from 150 ng RNA) either for miR or for mRNA analysis was used for real‐time PCR analysis. The experimental settings were as previously described.^4^, ^5^, ^6^ All samples (Vim3 and Mxi‐2) were normalized to ß‐actin as reference gene. All experiments were done in triplicate. When working with miRs, instead of the ß‐actin, 5 s rRNA was used for normalization. The ΔΔC_T_ method was used for calculation as outlined in User Bulletin 2 (PE Applied Biosystems, Forster City, USA). All qRT‐PCR was performed according to the SYBR™ Green protocol (Thermo Fisher Scientific, Warrington, UK). Untreated cells were used as controls. For the statistical significance of qRT‐PCR values, Student's t‐test was applied. (Table 1)

### 2.8 Statistical analysis

The GraphPad Prism 5 (San Diego, California, USA) program was used for statistical analysis. Analysis of variance (ANOVA) was performed, and the significant differences were calculated (*p < 0.05, **p < 0.01, and ***p < 0.001). All differences without stars were not of statistically significant.

## 3 RESULTS

**FIGURE 1:**
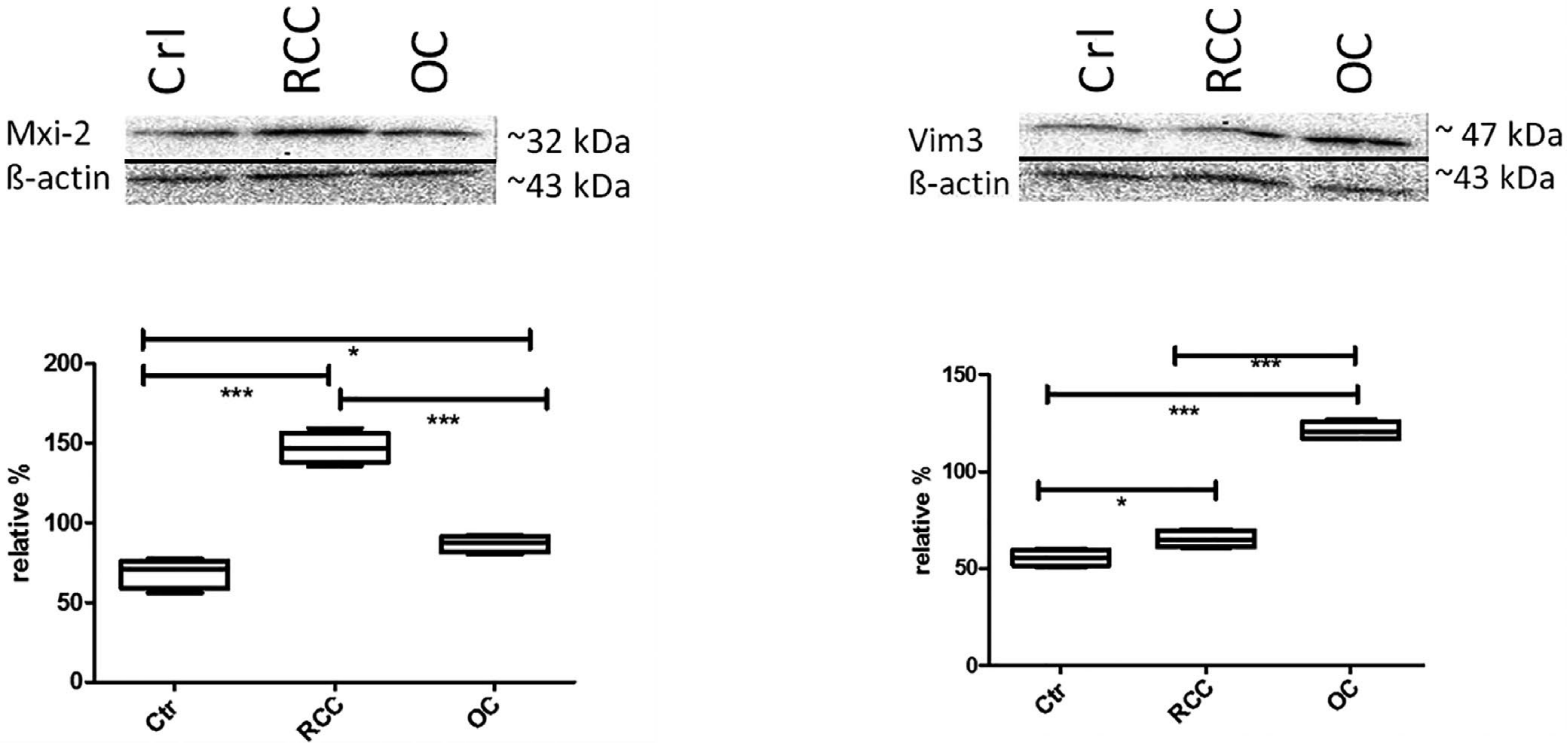
Western blot results of RCC, oncocytoma, and control urine samples for the detection of Vim3 and Mxi‐2. For analysis, 20 µg of total protein was loaded (*p < 0.05, **p < 0.01, and ***p < 0.001)

As previously shown in Brandenstein et al. 2018, both miRs were detectable in urine samples correlating with the tumor entity as published in von Brandenstein et al. 2018 and.^1^, ^7^ Vim3 was upregulated in oncocytoma, whereas Mxi‐2 was upregulated in RCCs. To analyze the target genes at the protein level, we performed a Western blot from sedimented urine samples. Here, it was possible to detect increased protein levels in both target sequences, namely Vim3 and Mxi‐2 in the corresponding kidney tumors, with significant differences (*p < 0.05, **p < 0.01, and ***p < 0.001) (Figure 1).

**FIGURE 2:**
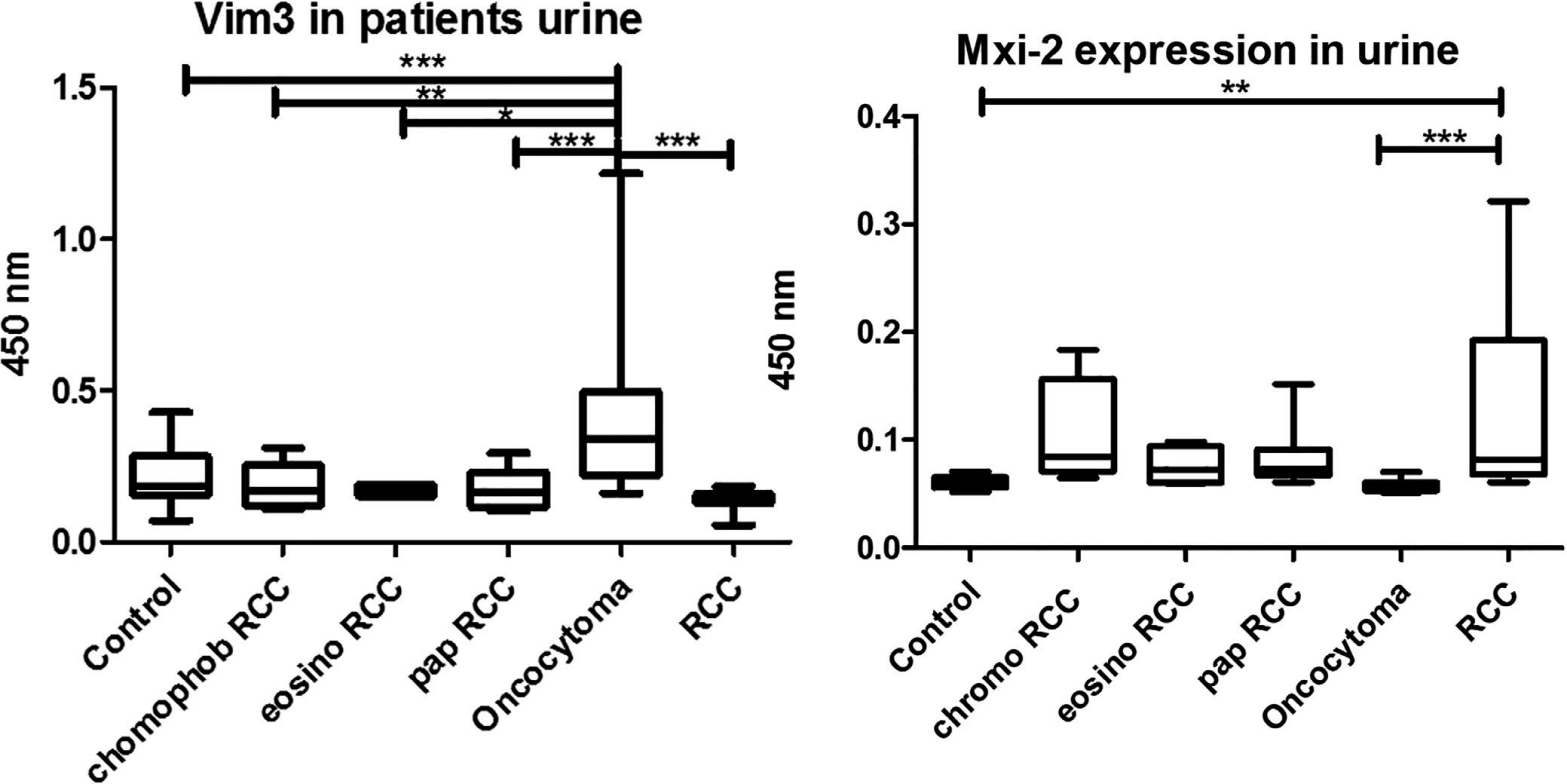
ELISA results of urine samples with different kidney tumor entities. Significant differences for Vim3 and Mxi‐2 expression were detectable (*p < 0.05, **p < 0.01, and ***p < 0.001)

Since it was possible to detect the protein levels in the Western blot, we performed the ELISA method as well. Figure 2 shows the results from ELISA test. Significantly increased urine levels of Vim3 were found in patients suffering from oncocytoma as compared to patients with RCC and the control group (***p < 0.0001) (sensitivity of 90.2% and specificity of 82.4%). In contrast, Mxi‐2 urine levels were significantly increased in patients with ccRCCs as compared to patients with an oncocytoma and the control group (p < 0.001).

**FIGURE 3:**
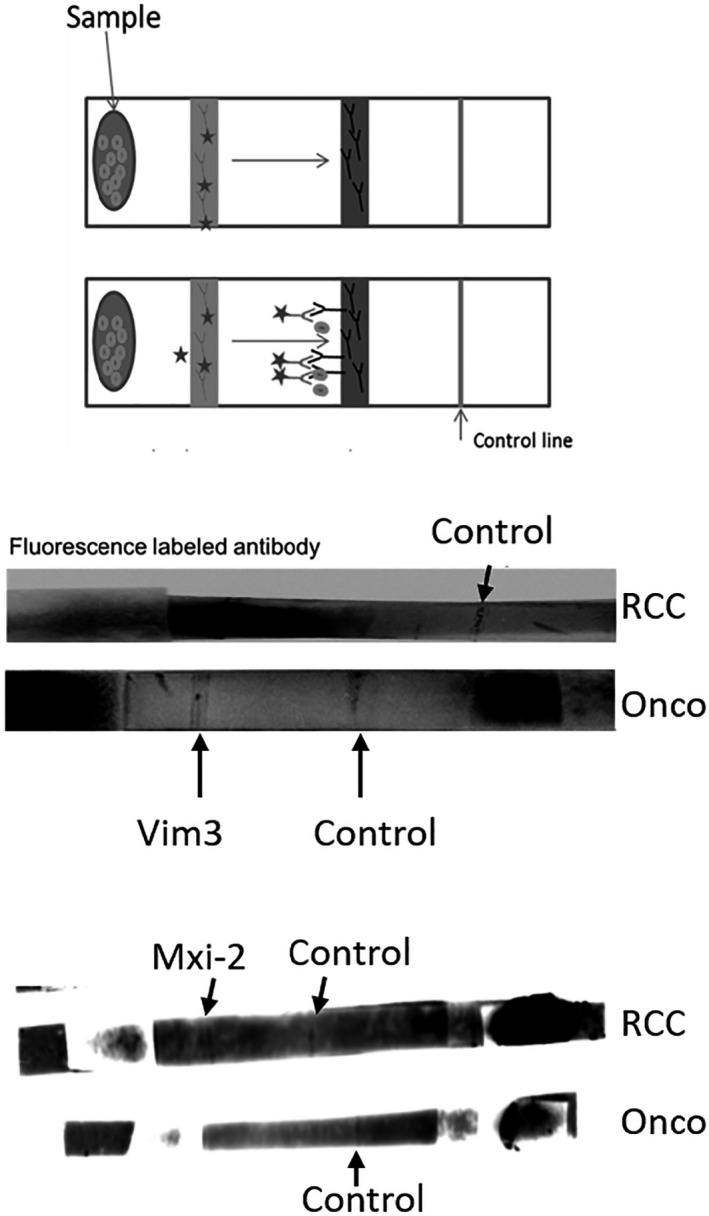
Exemplification of the performed lateral flow assay (upper part). Lower part demonstrates the Vim3 signal in oncocytoma urine sample and the corresponding negative control with RCC urine

For the faster analysis of urines, a lateral flow assay for the detection of Vim3 was also designed. Figure 3 illustrates the lateral flow assay that was performed for the detection of Vim3 in the patients’ urine.

**FIGURE 4:**
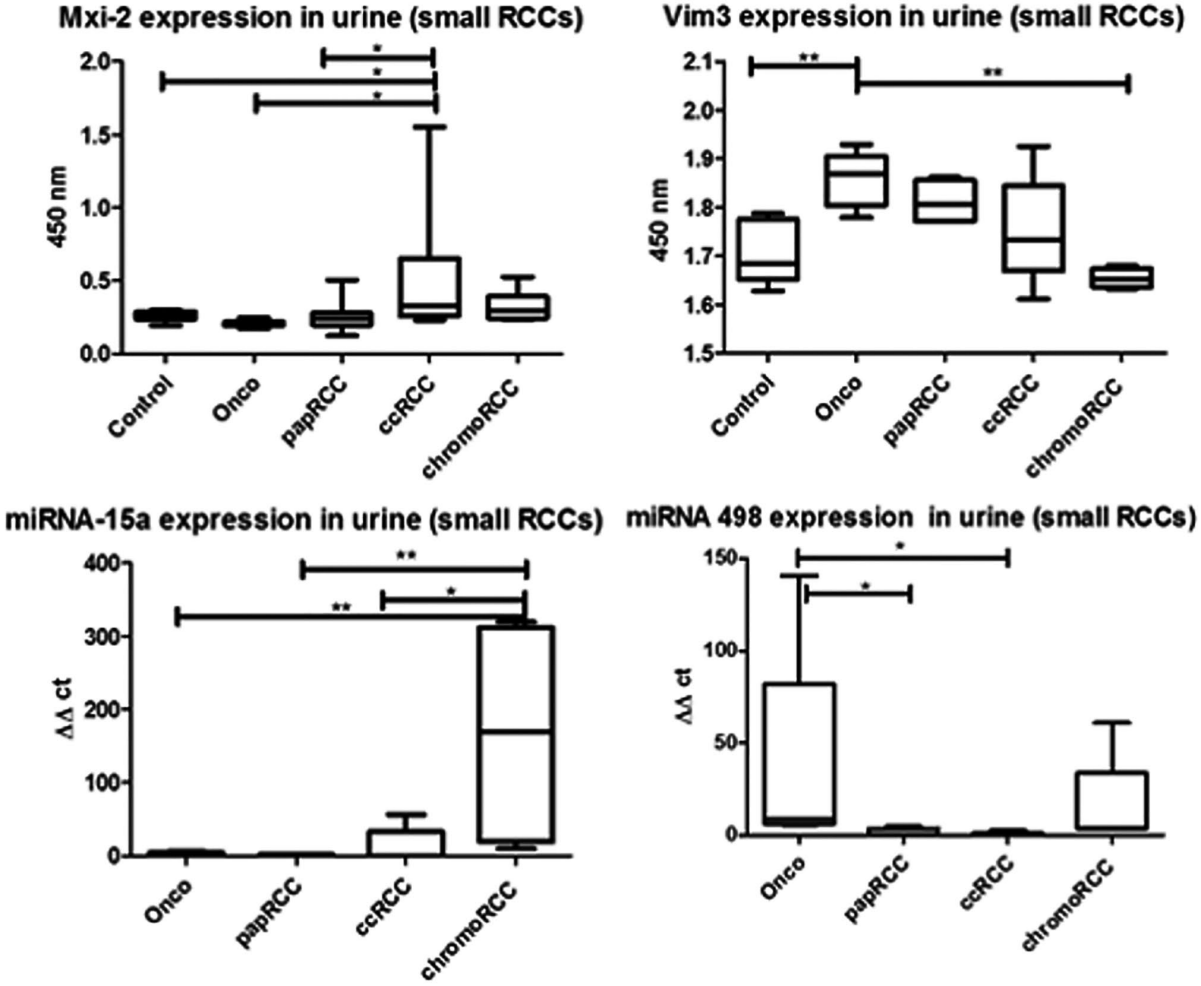
ELISA results of urine samples with different small kidney tumor entities (<4 cm). Significant differences for Vim3 and Mxi‐2 expression were detectable (*p < 0.05, **p < 0.01, and ***p < 0.001). qRT‐PCR results showing similar significances

As shown, it was possible to generate an uptake of the Vim3 signal in the urine sample of patients with diagnosed oncocytoma in comparison with patients suffering from RCC. The combined analysis of miR‐15a/Mxi‐2 and miR‐498/Vim3 in small kidney cancers (<4 cm) shows equal results as above mentioned (Figure 4).

## 4 DISCUSSION

There is a great demand for non‐invasive diagnostic tools of renal masses in the clinical routine. About 3–7% of renal tumors in adults are classified as benign renal oncocytoma.^8^ The frequency of benign histology in small renal masses (SRMS), that is, <4 cm in diameter, is 20–30%..^9^ Although most of SRMS undergo percutaneous biopsy prior to any procedure, such as nephron sparing surgery or active surveillance, 12–20% of biopsies are inconclusive.^10^ Any non‐invasive test with a highly reliable sensitivity and specificity to differentiate between benign and malignant lesions would help to reduce the frequency of overtreatment. These tumors are mostly treated by organ sparing surgery or active surveillance. Because radiological assessment of whether the renal mass is a benign oncocytoma or a malignant RCC is not safely possible,^11^ further non‐invasive diagnostic tools are urgently needed to avoid unnecessary surgeries. There is the possibility that our results demonstrate a feasible solution. We were able to identify two proteins for the non‐invasive differentiation of kidney tumors. Vim3 and Mxi‐2 urine levels show a clear correlation to the specific tumor entity. Vim3 is upregulated in urine samples from patients with oncocytoma and correlates with increased miR‐498 levels. We believe Vim3 to be a useful diagnostic tool as a biomarker for the differentiation of benign oncocytoma and malignant RCCs in patients’ urine. These promising findings should be further validated in a prospective clinical trial. We also found that Mxi‐2 is only upregulated in RCC urine samples and correlates with the increased miR‐15a levels found in urine samples of patients with RCC. Both proteins were tumor‐sized and independently upregulated as exemplified in Table 2. As mentioned earlier, both miRs are responsible for the production of the C‐terminal truncated proteins Vim3 and Mxi‐2, so we can assume that we managed to transfer the former findings of increased miR levels to the protein level.

As protein diagnostics are significantly faster and more economical than miR tools, our procedure presents a significant step toward biomarker‐supported diagnosis of renal masses. This is especially true in the realm of clinical routine and financing. Beyond this, the pre‐surgical differentiation between benign and malignant kidney tumors based on the cross‐sectional imaging fails to diagnose benign masses in more than 20% of all small lesions (<4 cm).^12^ Therefore, a non‐invasive test for the differentiation between benign and malignant RCCs is of great importance, especially with a view toward preventing the overtreatment of patients. Nevertheless, two other urine markers are also reported to be upregulated in urine samples, that is, aquaporin 1 and perilipin 2. These two markers can differentiate the clear cell and papillary RCC subtypes with a high sensitivity and specificity ^13^; however, to date no marker for the differentiation between benign and malignant kidney tumors is available.

Using the highly specific, non‐invasive urine markers, Vim3 and Mxi‐2, we would be able to fill the gap and reduce the number of overtreated patients as well as the number of unnecessary surgeries. Furthermore, even the detection as well as pre‐surgical differentiation in small kidney cancers is possible with the measurement of the two predicted miRs as well as the proteins.

## AUTHOR CONTRIBUTIONS

MvB performed the experimental design and statistical analysis and developed the theory. JH collected the urine samples and involved in result discussion. BK organized the samples and performed the experiments. TN discussed the results and performed the statistical analysis. MH performed ELISAs and miR isolation. AH and JWUF developed the theory and supervised the project. AH, JWUF, and MvB wrote the article with input from all authors.

## Data Availability

The datasets analyzed during the current study are available from the corresponding author on reasonable request.
